# Supplementary figures and images for: Exposure to dexamethasone modifies transcriptomic responses of free-living stages of Strongyloides stercoralis
Source: PLoS One. 2021 Jun 28;16(6):e0253701. doi: 10.1371/journal.pone.0253701 (PMC8238218; doi:10.1371/journal.pone.0253701)

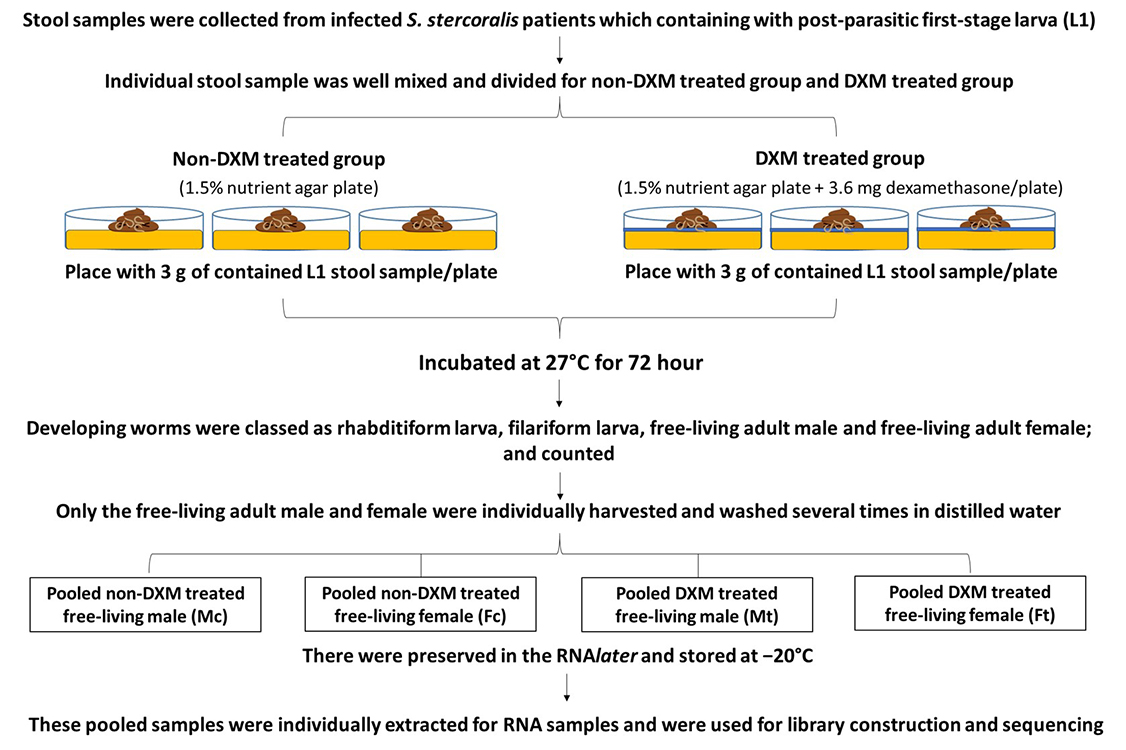

Supplement: S1 Fig — (TIF) [file pone.0253701.s001.tif]

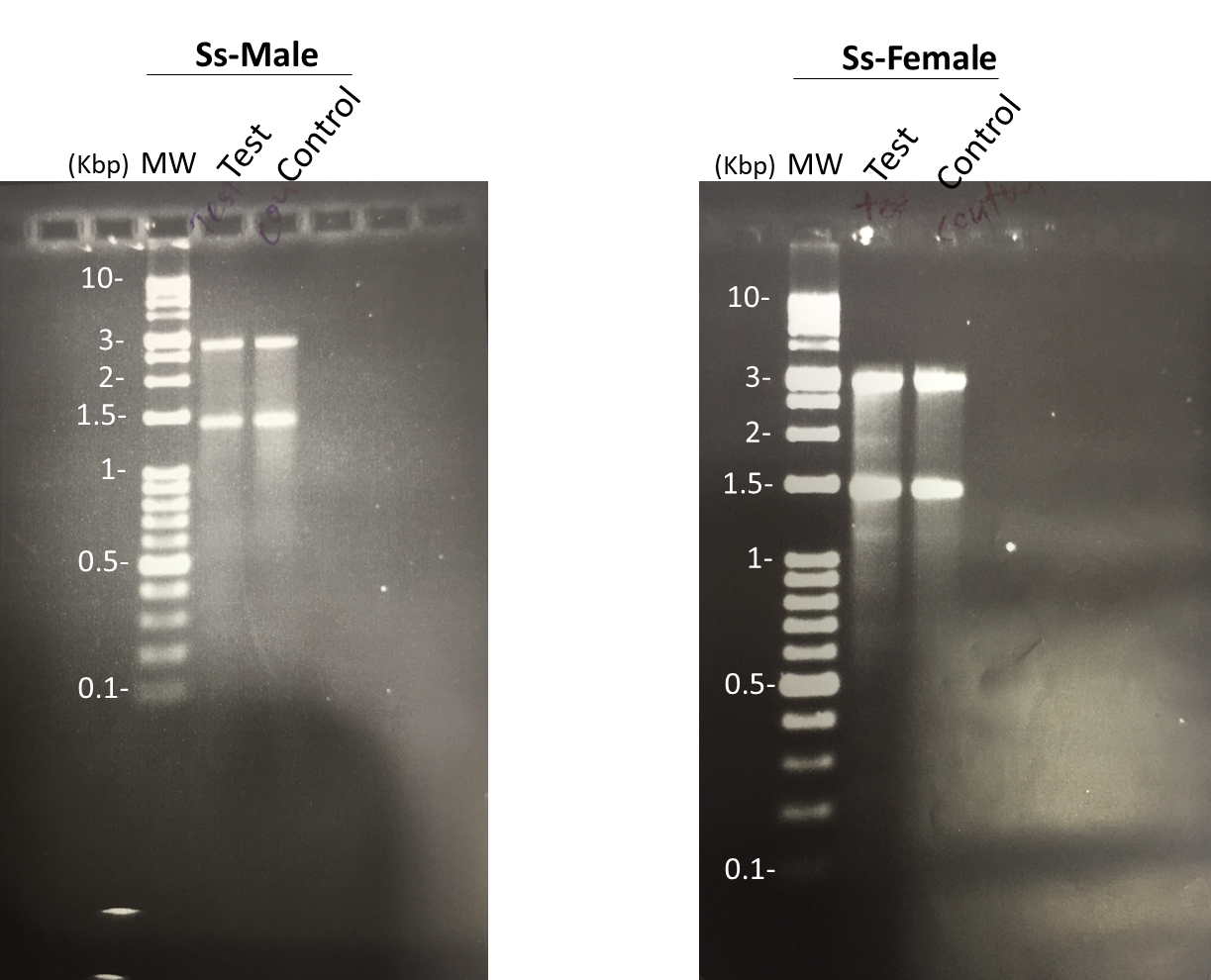

Supplement: S2 Fig — Left, the total RNA pattern extracted from pooled free-living adult male control (Control), free-living adult male treated with DXM (Test). Right, the total RNA pattern extracted free-living adult female control (Control)and free-living adult female treated with DXM (Test). MW, DNA ladder marker. (TIF) [file pone.0253701.s002.tif]

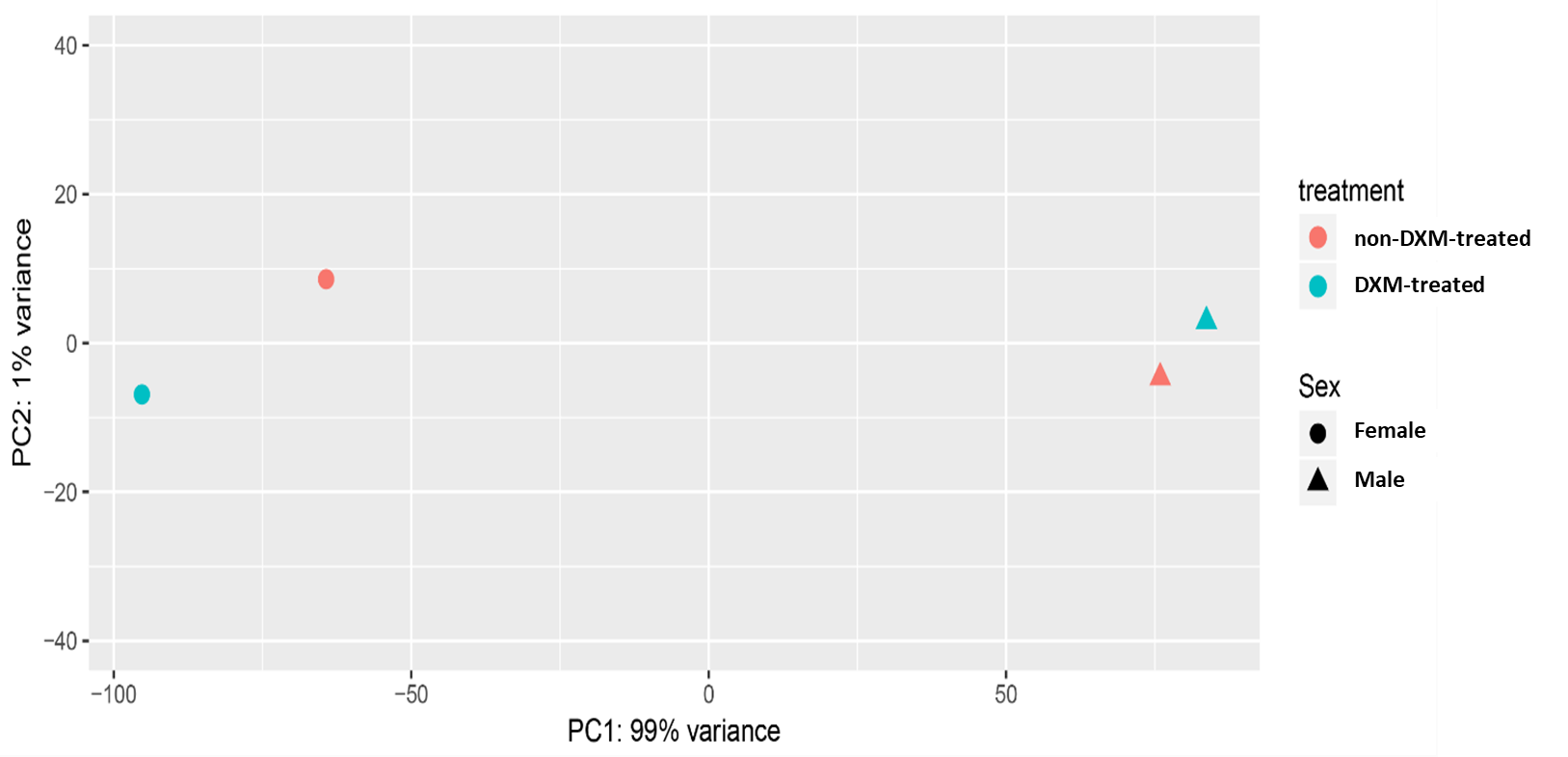

Supplement: S3 Fig — (TIF) [file pone.0253701.s003.tif]

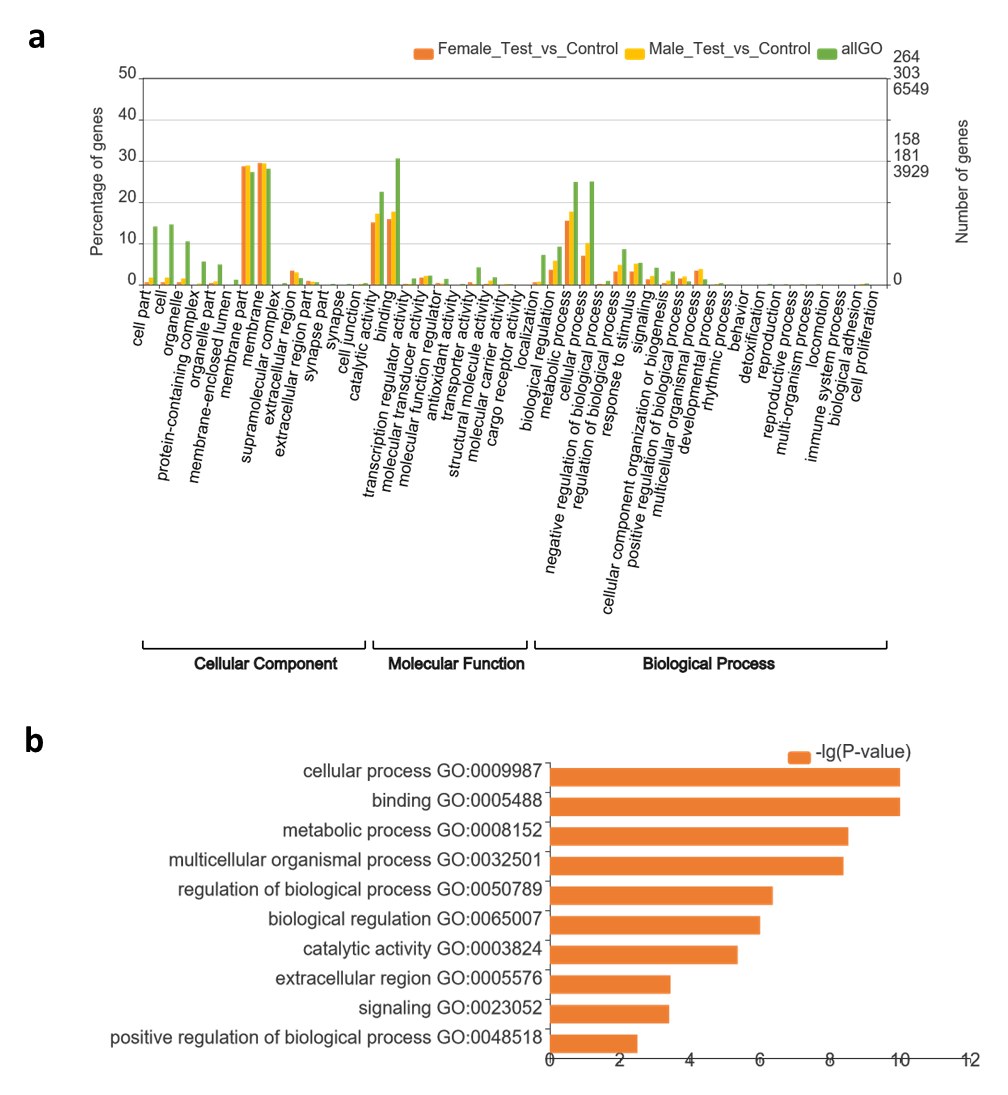

Supplement: S4 Fig — (a) The WEGO histogram of Fc versus Ft and Mc versus Mt targeted DEGs. The x-axis displays the GO terms. The right y-axis shows the gene numbers, while the left y-axis shows the percentages. (b) Log of P-values of GO terms indicated the significant differences between the down and up-regulated genes. (TIF) [file pone.0253701.s004.tif]
