# Supplementary material for: Exposure to dexamethasone modifies transcriptomic responses of free-living stages of Strongyloides stercoralis
Source: PLoS One. 2021 Jun 28;16(6):e0253701. doi: 10.1371/journal.pone.0253701 (PMC8238218; doi:10.1371/journal.pone.0253701)
Supplement: S3 Table — (PDF) [file pone.0253701.s008.pdf]

S3 Table. The top 30 differentially expressed genes by cluster analysis base on the reference genome mapping analysis.

List of 30 top Up-regulated expression gene in Female control Vs Female Test

Gene ID	GOID	GO term name	Gene description	UniProtKB entry	*C. elegans* orthologue
SSTP_0000443100	GO:0000723	telomere maintenance	ATP-dependent DNA helicase	A0A0K0E4L2	-
	GO:0003678	DNA helicase activity			
	GO:0006281	DNA repair			
	GO:0000166	nucleotide binding			
	GO:0005524	ATP binding			
	GO:0016787	hydrolase activity			
	GO:0006974	cellular response to DNA damage stimulus			
	GO:0004386	helicase activity			
	GO:0032508	DNA duplex unwinding			
	GO:0006310	DNA recombination			
SSTP_0000064900	GO:0004523	RNA-DNA hybrid ribonuclease activity	RNase H domain-containing protein	A0A0K0DTT6	-
	GO:0003676	nucleic acid binding			
	GO:0090502	RNA phosphodiester bond hydrolysis, endonucleolytic			
SSTP_0000962500	-	-	Uncharacterized protein	A0A0K0EJH9	***dyla-1*** (Dynein Light chain, Axonemal p28 type)
SSTP_0001197500	-	-	Uncharacterized protein	A0A0K0ER90	-
SSTP_0000684400	-	-	-	-	-
SSTP_0001192600	GO:0007186	G protein-coupled receptor signaling pathway	G_PROTEIN_RECEP_F1_2 domain-containing protein	A0A0K0ER44	***gnrr-7*** (human Gonadotropin-Releasing hormone Receptor (GnRHR) related)
	GO:0004930	G protein-coupled receptor activity			

	GO:0016021	integral component of membrane			
	GO:0016020	membrane			
SSTP_0000228300	GO:1902884	positive regulation of response to oxidative stress	Uncharacterized protein	A0A0K0DYG7	-
	GO:0030206	chondroitin sulfate biosynthetic process			
	GO:0047756	chondroitin 4-sulfotransferase activity			
	GO:0016020	membrane			
	GO:0016021	integral component of membrane			
SSTP_0001113000	GO:0016757	transferase activity, transferring glycosyl groups	Uncharacterized protein	A0A0K0ENU3	-
	GO:0016020	membrane			
SSTP_0000087700	-	-	Uncharacterized protein	A0A0K0DUG4	-
SSTP_0000048700	GO:1902884	positive regulation of response to oxidative stress	Uncharacterized protein	A0A0K0DTC4	***Y48G1BL.7, B0273.115*** (Is predicted to have chondroitin 4-sulfotransferase activity)
	GO:0030206	chondroitin sulfate biosynthetic process			
	GO:0047756	chondroitin 4-sulfotransferase activity			
	GO:0008146	sulfotransferase activity			
	GO:0016021	integral component of membrane			
	GO:0016020	membrane			
SSTP_0000612200	-	-	Uncharacterized protein	A0A0K0E9D9	-
SSTP_0000405600	-	-	Uncharacterized protein	A0A0K0E3I4	-
SSTP_0000661000	GO:0004222	metalloendopeptidase activity	Metalloendopeptidase	A0A0K0EAT5	***nas-12*** (Nematode Astacin protease)
	GO:0008237	metallopeptidase activity			
	GO:0006508	proteolysis			

	GO:0008270	zinc ion binding			
	GO:0046872	metal ion binding			
	GO:0016787	hydrolase activity			
	GO:0008233	peptidase activity			
SSTP_0000847800	-	-	SCP domain-containing protein	A0A0K0EG68	-
SSTP_0000001300	-	-	Uncharacterized protein	A0A0K0DRZ4	-
SSTP_0000386400	GO:0016020	membrane	Uncharacterized protein	A0A0K0E2Z5	-
	GO:0016021	integral component of membrane			
SSTP_0001038966	GO:0003676	nucleic acid binding	Uncharacterized protein	A0A0K0ELP8	-
SSTP_0000430400	GO:0016020	membrane	Uncharacterized protein	A0A0K0E485	-
	GO:0016021	integral component of membrane			
SSTP_0001095700	GO:0016020	membrane	Uncharacterized protein	A0A0K0ENC0	-
	GO:0016021	integral component of membrane			
SSTP_0000356800	GO:0016020	membrane	Uncharacterized protein	A0A0K0E247	-
	GO:0016021	integral component of membrane			
SSTP_0000108400	GO:0016020	membrane	Uncharacterized protein	A0A0K0DV17	-
	GO:0016021	integral component of membrane			
SSTP_0000277300	GO:0004523	RNA-DNA hybrid ribonuclease activity	Uncharacterized protein	A0A0K0DZW5	-
	GO:0015074	DNA integration			
	GO:0003676	nucleic acid binding			
	GO:0090502	RNA phosphodiester bond hydrolysis, endonucleolytic			
SSTP_0001111700	GO:0008417	fucosyltransferase activity	Glyco_tran_10_N domain-containing protein	A0A0K0ENT0	***fut-3*** (Fucosyltransferase)
	GO:0006486	protein glycosylation			

	GO:0016020	membrane			
	GO:0016740	transferase activity			
	GO:0016021	integral component of membrane			
	GO:0005794	Golgi apparatus			
	GO:0016757	transferase activity, transferring glycosyl groups			
	GO:0032580	Golgi cisterna membrane			
	GO:0036065	fucosylation			
SSTP_0000328900	GO:0003779	actin binding	Uncharacterized protein	A0A0K0E1C4	-
SSTP_0001168000	GO:0016020	membrane	Uncharacterized protein	A0A0K0EQF1	
	GO:0016021	integral component of membrane			
SSTP_0000637800	GO:0004521	endoribonuclease activity	XendoU domain-containing protein	A0A0K0EA51	***endu-1*** (Endonuclease, poly(U) specific)
	GO:0090502	RNA phosphodiester bond hydrolysis, endonucleolytic			
SSTP_0001014700	GO:1902884	positive regulation of response to oxidative stress	Uncharacterized protein	A0A0K0EL03	-
	GO:0030206	chondroitin sulfate biosynthetic process			
	GO:0047756	chondroitin 4-sulfotransferase activity			
	GO:0008146	sulfotransferase activity			
	GO:0016021	integral component of membrane			
SSTP_0001166900	GO:0016020	membrane	Uncharacterized protein	A0A0K0EQE0	
	GO:0016021	integral component of membrane			
SSTP_0001118700	GO:0004222	metalloendopeptidase activity	Metalloendopeptidase	A0A0K0EP00	-
	GO:0008237	metallopeptidase activity			
	GO:0006508	proteolysis			

	GO:0008270	zinc ion binding			
	GO:0046872	metal ion binding			
	GO:0016787	hydrolase activity			
	GO:0008233	peptidase activity			
SSTP_0000564300	GO:0016020	membrane	Uncharacterized protein	A0A0K0E813	-
	GO:0016021	integral component of membrane			

List of 30 top Down-regulated expression gene in Female control Vs Female Test

Gene ID	GOID	GO term name	Gene description	UniProtKB entry	*C. elegans* orthologue
SSTP_0001151800	-	-	Uncharacterized protein	A0A0K0EPY9	-
SSTP_0000505100	-	-	Uncharacterized protein	A0A0K0E6C0	-
SSTP_0001222900	GO:0006915	apoptotic process	Uncharacterized protein	A0A0K0ES00	-
	GO:0016020	membrane			
	GO:0016021	integral component of membrane			
SSTP_0001102300	-	-	Uncharacterized protein	A0A0K0ENI6	-
SSTP_0000237200	GO:0003723	RNA binding	Uncharacterized protein	A0A0K0DYQ6	-
SSTP_0000772800	GO:0046983	protein dimerization activity	BHLH domain-containing protein	A0A0K0EE14	***hlh-15*** (Helix Loop Helix)
SSTP_0000908600	GO:0016020	membrane	Uncharacterized protein	A0A0K0EHY1	-
	GO:0016021	integral component of membrane			
SSTP_0000504100	-	-	Uncharacterized protein	A0A0K0E6B0	-
SSTP_0000115400	GO:0018996	molting cycle, collagen and cuticulin-based cuticle	Zinc metalloproteinase	A0A0K0DV88	-
	GO:0004222	metalloendopeptidase activity			
	GO:0008237	metallopeptidase activity			
	GO:0006508	proteolysis			
	GO:0008270	zinc ion binding			
	GO:0046872	metal ion binding			

	GO:0016787	hydrolase activity			
	GO:0008233	peptidase activity			
	GO:0005576	extracellular region			
SSTP_0001115210	GO:0004222	metalloendopeptidase activity	Metalloendopeptidase	A0A0K0ENW5	-
	GO:0008237	metallopeptidase activity			
	GO:0006508	proteolysis			
	GO:0008270	zinc ion binding			
	GO:0046872	metal ion binding			
	GO:0016787	hydrolase activity			
	GO:0008233	peptidase activity			
SSTP_0001111300	-	-	Uncharacterized protein	A0A0K0ENS6	-
SSTP_0000040650	-	-	Uncharacterized protein	A0A0K0DT46	-
SSTP_0000811250	-	-	Uncharacterized protein	A0A0K0EF49	-
SSTP_0000648800	GO:0016020	membrane	Uncharacterized protein	A0A0K0EAG3	-
	GO:0016021	integral component of membrane			
SSTP_0001113200	-	-	Uncharacterized protein	A0A0K0ENU5	-
SSTP_0000450700	-	-	Uncharacterized protein	A0A0K0E4T7	-
SSTP_0000828800	GO:0016020	membrane	Uncharacterized protein	A0A0K0EFM7	-
	GO:0016021	integral component of membrane			
SSTP_0001051400	GO:0003924	GTPase activity	Uncharacterized protein	A0A0K0EM26	***T26C12.3***
	GO:0007165	signal transduction			
	GO:0005525	GTP binding			
	GO:0016020	membrane			
	GO:0000166	nucleotide binding			
SSTP_0000700600	-	-	Uncharacterized protein	A0A0K0EM26	-
SSTP_0000109500	GO:0008080	N-acetyltransferase activity	N-acetyltransferase domain-containing protein	A0A0K0DV28	***daf-31*** (abnormal Dauer Formation)

SSTP_0000992300	GO:0016020<br>GO:0016021	membrane<br>integral component of membrane	Uncharacterized protein	A0A0K0EKC9	-
SSTP_0000365300	GO:0006468<br>GO:0004672<br>GO:0005524<br>GO:0000166<br>GO:0004674	protein phosphorylation<br>protein kinase activity<br>ATP binding<br>nucleotide binding<br>protein serine/threonine kinase activity	Protein kinase domain-containing protein	A0A0K0E2D3	-
SSTP_0000246700	-	-	Uncharacterized protein	A0A0K0DZ01	-
SSTP_0000425700	GO:0007186<br>GO:0004930<br>GO:0016021<br>GO:0016020	G protein-coupled receptor signaling pathway<br>G protein-coupled receptor activity<br>integral component of membrane<br>membrane	Uncharacterized protein	A0A0K0E438	***srt-48, srt-45***, ***srt-49***, ***Y57G11C.28, srt-50*** (Serpentine Receptor, class T) Is affected by several genes including ***daf-2, srt-44, srt-43, srt-47*** (Serpentine Receptor, class T), ***Y57G11C.30***
SSTP_0000290300	-	-	Uncharacterized protein	A0A0K0E089	-
SSTP_0001108500	GO:0070008<br>GO:0008236<br>GO:0004252<br>GO:0006508	serine-type exopeptidase activity<br>serine-type peptidase activity<br>serine-type endopeptidase activity<br>proteolysis	Uncharacterized protein	A0A0K0ENP8	-
SSTP_0000202400	GO:0036459<br>GO:0016579<br>GO:0006511	thiol-dependent ubiquitinyl hydrolase activity<br>protein deubiquitination<br>ubiquitin-dependent protein catabolic process	USP domain-containing protein	A0A0K0DXQ9	***math-33*** (MATH (meprin-associated Traf homology) domain containing), Is an ortholog of human USP7 (ubiquitin specific peptidase 7).
SSTP_0000632600	GO:0016020	membrane	Uncharacterized protein	A0A0K0E9Z4	-

	GO:0016021	integral component of membrane			
SSTP_0000074488	-	-	Uncharacterized protein	A0A0K0DU31	-
SSTP_0000303850	-	-	Uncharacterized protein	A0A0K0E0M6	-

List of 30 top Up-regulated expression gene in Male control Vs Male Test

Gene ID	GOID	GO term name	Gene description	UniProtKB entry	*C. elegans* orthologue
SSTP_0001269800	GO:0004190	aspartic-type endopeptidase activity	Uncharacterized protein	A0A0K0ETC0	-
	GO:0003676	nucleic acid binding			
	GO:0006508	proteolysis			
	GO:0008270	zinc ion binding			
SSTP_0000737000	GO:0016705	oxidoreductase activity, acting on paired donors, with incorporation or reduction of molecular oxygen	Uncharacterized protein	A0A0K0ED06	***cyp-29A2*** (Cytochrome P450 family)
	GO:0005506	iron ion binding			
	GO:0020037	heme binding			
	GO:0055114	oxidation-reduction process			
	GO:0046872	metal ion binding			
	GO:0016491	oxidoreductase activity			
	GO:0004497	monooxygenase activity			
SSTP_0001222300	GO:0005615	extracellular space	Uncharacterized protein	A0A0K0ED06	-
SSTP_0000274900	-	-	Uncharacterized protein	A0A0K0DZT8	-
SSTP_0000507400	-	-	Uncharacterized protein	A0A0K0E6E3	-
SSTP_0000443100	GO:0000723	telomere maintenance	ATP-dependent DNA helicase	A0A0K0E4L2	-
	GO:0003678	DNA helicase activity			
	GO:0006281	DNA repair			

	GO:0000166	nucleotide binding			
	GO:0005524	ATP binding			
	GO:0016787	hydrolase activity			
	GO:0006974	cellular response to DNA damage stimulus			
	GO:0004386	helicase activity			
	GO:0032508	DNA duplex unwinding			
	GO:0006310	DNA recombination			
SSTP_0000198700	GO:0016020	membrane	Uncharacterized protein	A0A0K0DXM2	-
	GO:0016021	integral component of membrane			
SSTP_0001116500	GO:0016020	membrane	PHB domain-containing protein	A0A0K0ENX8	***sto-1*** (Stomatin)
	GO:0016021	integral component of membrane			
SSTP_0001129500	-	-	Uncharacterized protein	A0A0K0EPA8	-
SSTP_0000845500	-	-	Uncharacterized protein	A0A0K0EG46	-
SSTP_0001116200	-	-	Lipoyl-binding domain-containing protein	A0A0K0ENX5	-
SSTP_0000455700	GO:0015074	DNA integration	Integrase catalytic domain-containing protein	A0A0K0E4Y9	
	GO:0003676	nucleic acid binding			
SSTP_0000512600	-	-	SCP domain-containing protein	A0A0K0E6J7	***scl-12, scl-13*** (SCP-Like extracellular protein)
SSTP_0000955800	GO:0004222	metalloendopeptidase activity	Metalloendopeptidase	A0A0K0EJB1	-
	GO:0008237	metallopeptidase activity			
	GO:0006508	proteolysis			
	GO:0046872	metal ion binding			
	GO:0016787	hydrolase activity			
	GO:0008233	peptidase activity			
SSTP_0000136600	GO:0035556	intracellular signal transduction	DEP domain-containing protein	A0A0K0DVV1	-
	GO:0007165	signal transduction			

SSTP_0000864200	-	-	Uncharacterized protein	A0A0K0EGN1	-
SSTP_0001212800	GO:0016020<br>GO:0016021	membrane<br>integral component of membrane	Uncharacterized protein	A0A0K0ERP8	***igcm-3*** (Immunoglobulin-like Cell adhesion Molecule family)
SSTP_0001051500	GO:0016020<br>GO:0016021<br>GO:0006811<br>GO:0005886<br>GO:0005921<br>GO:0030054	membrane<br>integral component of membrane<br>ion transport<br>plasma membrane<br>gap junction<br>cell junction	Innexin	A0A0K0EM27	-
SSTP_0001247300	GO:0016020<br>GO:0016021	membrane<br>integral component of membrane	Uncharacterized protein	A0A0K0ESP4	-
SSTP_0001113500	GO:0016740<br>GO:0016757	transferase activity<br>transferase activity, transferring glycosyl groups	Glycosyltransferase family 92 protein	A0A0K0ENU8	-
SSTP_0000031625	GO:0016020<br>GO:0016021	membrane<br>integral component of membrane	Uncharacterized protein	A0A0K0DSV3	-
SSTP_0001172700	-	-	Uncharacterized protein	A0A0K0EQJ8	-
SSTP_0001095700	GO:0016020<br>GO:0016021	membrane<br>integral component of membrane	Uncharacterized protein	A0A0K0ENC0	-
SSTP_0000012500	GO:1902884<br>GO:0030206<br>GO:0047756<br>GO:0008146<br>GO:0016021	positive regulation of response to oxidative stress<br>chondroitin sulfate biosynthetic process<br>chondroitin 4-sulfotransferase activity<br>sulfotransferase activity<br>integral component of membrane	Uncharacterized protein	A0A0K0DSB5	-

SSTP_0000877200	-	-	Cytochrome b5 heme-binding domain-containing protein	A0A0K0EH15	-
SSTP_0000060400	GO:0046983	protein dimerization activity	BHLH domain-containing protein	A0A0K0DTP1	***hlh-13*** (Helix Loop Helix)
SSTP_0000835200	GO:0016020<br>GO:0016021	membrane<br>integral component of membrane	Uncharacterized protein	A0A0K0EFU5	-
SSTP_0000830900	-	-	Uncharacterized protein	A0A0K0EFP9	-
SSTP_0000490600	-	-	Uncharacterized protein	A0A0K0E5X1	-
SSTP_0001018100	-	-	Uncharacterized protein	A0A0K0EL37	-

List of 30 top Down-regulated expression gene in Male control Vs Male Test

Gene ID	GOID	GO term name	Gene description	UniProtKB entry	*C. elegans* orthologue
SSTP_0000870600	GO:0042302<br>GO:0016020<br>GO:0016021	structural constituent of cuticle<br>membrane<br>integral component of membrane	Col_cuticle_N domain-containing protein	A0A0K0EGU6	-
SSTP_0001127700	GO:0018996<br>GO:0004222<br>GO:0008237<br>GO:0006508<br>GO:0008270<br>GO:0005576	molting cycle, collagen and cuticulin-based cuticle<br>metalloendopeptidase activity<br>metallopeptidase activity<br>proteolysis<br>zinc ion binding<br>extracellular region	Zinc metalloproteinase	A0A0K0EP91	***nas-31*** (Nematode Astacin protease)
SSTP_0000530600	GO:0006508<br>GO:0008270<br>GO:0004181	proteolysis<br>zinc ion binding<br>metallocarboxypeptidase activity	Peptidase_M14 domain-containing protein	A0A0K0E730	-
SSTP_0000580200	GO:0003676	nucleic acid binding	C2H2-type domain-containing protein	A0A0K0E8H1	-
SSTP_0000259900	-	-	Uncharacterized protein	A0A0K0DZD4	-
SSTP_0001098400	GO:0042302	structural constituent of cuticle	Col_cuticle_N domain-containing protein	A0A0K0ENE7	-

	GO:0016020	membrane			
	GO:0016021	integral component of membrane			
SSTP_0000419500	-	-	Uncharacterized protein	A0A0K0E3X5	-
SSTP_0000020700	-	-	Uncharacterized protein	A0A0K0DSJ5	-
SSTP_0001103800	-	-	Uncharacterized protein	A0A0K0ENK1	-
SSTP_0000246300	-	-	Uncharacterized protein	A0A0K0DYZ7	-
SSTP_0000061600	GO:0007186	G protein-coupled receptor signaling pathway	G_PROTEIN_RECEP_F1_2 domain-containing protein	A0A0K0DTQ2	***npr-35*** (Neuropeptide Receptor family)
	GO:0004930	G protein-coupled receptor activity			
	GO:0016021	integral component of membrane			
	GO:0016020	membrane			
	GO:0007165	signal transduction			
SSTP_0000223400	GO:0006486	protein glycosylation	Uncharacterized protein	A0A0K0DYC0	***E03H4.3, T09E11.12 pseudogene, F56H6.1, C17A2.3, C02H6.1***
	GO:0008378	galactosyltransferase activity			
	GO:0016020	membrane			
	GO:0016021	integral component of membrane			
SSTP_0000380400	-	-	Uncharacterized protein	A0A0K0E2T5	***mltn-12*** (Mlt-Ten (mlt-10) related) (Moulting cycle MLT-10-like protein)
SSTP_0000984200	GO:0042773	ATP synthesis coupled electron transport	Proton_antipo_M domain-containing protein	A0A0K0EK50	***nduo-4*** (mitochondrial genome encoded NADH-Ubiquinone Oxidoreductase chain homolog)
	GO:0008137	NADH dehydrogenase (ubiquinone) activity			
	GO:0055114	oxidation-reduction process			
	GO:0016020	membrane			
	GO:0016021	integral component of membrane			

SSTP_0000353600	-	-	RT_RNaseH domain-containing protein	A0A0K0E216	-
SSTP_0000449800	GO:0005515<br>GO:0016020<br>GO:0016021	protein binding<br>membrane<br>integral component of membrane	Uncharacterized protein	A0A0K0E4S8	***egg-6*** (EGG sterile (unfertilizable))
SSTP_0000688300	-	-	Uncharacterized protein	A0A0K0EBL1	-
SSTP_0001102300	-	-	Uncharacterized protein	A0A0K0ENI6	-
SSTP_0000210100	GO:0007606<br>GO:0004888<br>GO:0016020<br>GO:0016021	sensory perception of chemical stimulus<br>transmembrane signaling receptor activity<br>membrane<br>integral component of membrane	Serpentine receptor class gamma	A0A0K0DXY7	-
SSTP_0000667700	-	-	Uncharacterized protein	A0A0K0EB06	-
SSTP_0000434300	-	-	Uncharacterized protein	A0A0K0E4C4	-
SSTP_0001227900	GO:0006470<br>GO:0004725<br>GO:0016311<br>GO:0008138<br>GO:0016791<br>GO:0016787<br>GO:0035335<br>GO:0004721	protein dephosphorylation<br>protein tyrosine phosphatase activity<br>dephosphorylation<br>protein tyrosine/serine/threonine phosphatase activity<br>phosphatase activity<br>hydrolase activity<br>peptidyl-tyrosine dephosphorylation<br>phosphoprotein phosphatase activity	Uncharacterized protein	A0A0K0ES51	***T12B3.1*** (protein tyrosine phosphatase domain containing 1)
SSTP_0000187300	GO:0031012	extracellular matrix	ZnMc domain-containing protein	A0A0K0DXA6	***Y50D7A.13***

	GO:0004222<br>GO:0008237<br>GO:0006508<br>GO:0008270<br>GO:0046872<br>GO:0016787<br>GO:0008233	metalloendopeptidase activity<br>metallopeptidase activity<br>proteolysis<br>zinc ion binding<br>metal ion binding<br>hydrolase activity<br>peptidase activity			
SSTP_0000385700	-	-	Uncharacterized protein	A0A0K0E2Y8	-
SSTP_0000447000	-	-	Uncharacterized protein	A0A0K0E4Q1	-
SSTP_0000261300	GO:0005576	extracellular region	SCP domain-containing protein	A0A0K0DZE9	***F09B9.5***
SSTP_0000488400	GO:0016020<br>GO:0016021<br>GO:0006914	membrane<br>integral component of membrane<br>autophagy	Autophagy_act_C domain-containing protein	A0A0K0E5V0	-
SSTP_0000965500	GO:0004222<br>GO:0008237<br>GO:0006508<br>GO:0008270<br>GO:0046872<br>GO:0016787<br>GO:0008233	metalloendopeptidase activity<br>metallopeptidase activity<br>proteolysis<br>zinc ion binding<br>metal ion binding<br>hydrolase activity<br>peptidase activity	Metalloendopeptidase	A0A0K0EJL1	-
SSTP_0001250500	GO:0016020<br>GO:0016021	membrane<br>integral component of membrane	Uncharacterized protein	A0A0K0ESS5	***Y11D7A.3, B0554.5, Y39D8A.1, ZK6.6, Y39B6A.29, ZK6.8, B0554.7, Y39B6A.27, Y37A1A.2***
SSTP_0001020300	-	-	Uncharacterized protein	A0A0K0EL59	-
